# Systematic review of antibiotic stewardship interventions for urinary tract infection management in the ambulatory setting

**DOI:** 10.1017/ash.2022.102

**Published:** 2022-05-16

**Authors:** Sonal Munsiff, Alexandra Sakai, Ghinwa Dumyati

## Abstract

**Background:** Urinary tract infections (UTIs) are common indications for antibiotics in ambulatory setting, and inappropriate use is prevalent. Fluoroquinolones account for 40% of antibiotics prescribed for uncomplicated UTIs, despite clinical guidance against their use as firstline agents. We conducted a systematic review to determine which antibiotic stewardship intervention(s) are effective in improving antibiotic prescribing for UTIs in the ambulatory setting. **Methods:** Following PRISMA guidelines, English-language literature from 1995 to September 21, 2021, was searched for articles about antimicrobial stewardship, UTI, and ambulatory setting from PubMed, Embase, and Central. Additional articles were identified from authors’ collections and references of pertinent articles. Studies were included if the authors implemented intervention targeting adults 18 years and older in outpatient setting (excluding emergency departments). Interventions were categorized into Guideline Development and Dissemination (GDD), Audit and Feedback, Clinical Decision Support System (CDSS), and Multimodal Interventions. **Results:** The literature search identified 1,899 papers; 14 papers were included in this review; and 4 additional papers were identified from other sources. The main interventions were GDD in 6 studies, audit and feedback in 3 studies, CDSS in 4 studies, and multimodal interventions in 5 stidues. These studies had heterogeneity of the practice settings and interventions. Moreover, 11 studies targeted primary care, 2 studies targeted urgent care, 1 study targeted both primary and urgent care, 2 studies were conducted in spinal cord injury clinics, and 2 studies were conducted in hospital-wide outpatient sites. Outcomes included (1) statistically significant increase in guideline-concordant antibiotic prescribing in 12 studies (range, 4.6%–246%); (2) statistically significant decrease in fluoroquinolone prescriptions (range, 9.1%–86.3%) in 7 of 9 studies focusing on fluoroquinolones; (3) significant decreases in drug resistance in urine pathogens in 2 studies that evaluated this. Provider education, in conjunction with passive CDSS tools, such as integrating order sets for UTI prescriptions with prefilled instructions into electronic medical records appeared most beneficial. Several studies have investigated negative impact and have found no increase in retreatment rates or worse outcomes. **Conclusions:** Our systematic literature review identified a limited number of studies with a variety of interventions that improved antibiotic use for UTIs in the ambulatory care setting. Provider education, in conjunction with CDSS tools, can be less time-consuming than audit and feedback and can target a large number of providers and practices. Future studies need to address sustainability over longer periods and should target specialty clinic populations because they have high burden of patients with multidrug-resistant UTI organisms.

**Funding:** None

**Disclosures:** None

